# The Swiss Multiple Sclerosis Registry (SMSR): study protocol of a participatory, nationwide registry to promote epidemiological and patient-centered MS research

**DOI:** 10.1186/s12883-018-1118-0

**Published:** 2018-08-13

**Authors:** Nina Steinemann, Jens Kuhle, Pasquale Calabrese, Jürg Kesselring, Giulio Disanto, Doron Merkler, Caroline Pot, Vladeta Ajdacic-Gross, Stephanie Rodgers, Milo Alan Puhan, Viktor von Wyl, the Swiss Multiple Sclerosis Registry, Bernd Anderseck, Pasquale Calabrese, Andrew Chan, Giulio Disanto, Britta Engelhardt, Claudio Gobbi, Roger Häussler, Christian P. Kamm, Susanne Kägi, Jürg Kesselring, Jens Kuhle, Roland Kurmann, Christoph Lotter, Kurt Luyckx, Doron Merkler, Patricia Monin, Stephanie Müller, Krassen Nedeltchev, Caroline Pot, Milo A. Puhan, Irene Rapold, Anke Salmen, Sven Schippling, Claude Vaney, Viktor von Wyl

**Affiliations:** 1 Epidemiology, Biostatistics and Prevention Institute, University of Zurich, Hirschengraben 84, CH-8001 Zurich, Switzerland; 2 Neurological Policlinic, University Hospital Basel, Basel, Switzerland; 3 Department of Psychology, University of Basel, Basel, Switzerland; 4 Rehabilitation Clinic Valens, Valens, Switzerland; 5 Department of Neurology, Regional Hospital Lugano (EOC), Lugano, Switzerland; 6 Division of Clinical Pathology, Geneva University Hospital, Geneva, Switzerland; 7 Department of Clinical Neuroscience, Centre hospitalier universitaire vaudois, Lausanne, Switzerland

**Keywords:** Multiple sclerosis, Health-related quality of life, Epidemiology, Switzerland, Patient-reported outcomes

## Abstract

**Background:**

Multiple sclerosis (MS) is one of the most frequently observed neurological conditions in Switzerland, but data sources for country-wide epidemiological trend monitoring are lacking. Moreover, while clinical and laboratory MS research are generally well established, there is a gap in patient-centered MS research to inform care management, or treatment decisions and policy making not only in Switzerland but worldwide.

**Methods:**

In light of these research gaps, the Swiss Multiple Sclerosis Society initiated and funded the Swiss Multiple Sclerosis Registry (SMSR) an open-ended, longitudinal and prospective, nationwide, patient-centered study.

The SMSR recruits adult persons with a suspected or confirmed MS diagnosis who reside or receive care in Switzerland. The SMSR has established a governance structure with clear rules and guidelines. It follows a citizen-science approach with direct involvement of persons with MS (PwMS), who contribute actively to registry development, operations, and research. Main scientific goals entail the study of MS epidemiology in Switzerland, health care access and provision, as well as life circumstances and wellbeing of persons with MS.

The innovative study design (“layer model”) offers several participation options with different time commitments. Data collection is by means of regular surveys and medical record abstraction. Survey participation is offered in different modes (web, paper & pencil) and in the three main national languages (German, French, Italian). Participants also receive regular data feedbacks for personal use and self-monitoring, contextualized in the whole population of study participants. Data feedbacks are also used to solicit data corrections of key variables from participants.

**Discussion:**

The SMSR combines the advantages of traditional and novel research methods in medical research and has recruited over 1600 PwMS in its first year. The future-oriented design and technology will enable a response not only to future technological innovations and research trends, but also to challenges in health care provision for MS.

**Trial registration:**

ClinicalTrials.gov NCT02980640; December 6, 2016; retrospectively registered.

## Background

Multiple sclerosis (MS) is the most common cause of non-traumatic disability among young adults in industrialized countries [1]. With an estimated 110 MS cases per 100,000 inhabitants, [2] the MS prevalence in Switzerland surpasses the median estimate for Europe - the region with the highest MS prevalence worldwide (80 per 100,000 inhabitants) [3]. However, the Swiss estimate has not been updated in nearly 30 years, mostly because no easily accessible routine data are available, for example from clinical care or health insurances. Unlike countries with national health care systems, the highly fragmented Swiss health care landscape suffers from a lack of standardization in data collection, an inexistent legal basis for mandatory reporting of severe chronic illnesses, as well as limited information technology (IT) system interoperability between different care and health insurance providers. In light of these constraints, the establishment of a medical registry with active data collection from various sources is one of very few viable options for establishing a long-term monitoring of epidemiological trends, but also for promoting personalized medicine approaches in MS [4].

As illustrated by a systematic survey of European MS registries, the role of registries in MS research is not limited to monitoring purposes [5]. The international MS registry landscape is very diverse, and many of these studies have made important contributions to MS epidemiology, treatment, diagnosis, and care research. However, several other disease aspects are less well covered by some existing registries, such as cost of illness, quality management of healthcare, patient preferences, and patient reported outcomes [5]. These research limitations can weigh heavy in chronic illnesses such as MS, often characterized by a complex management, an unclear evidence base for treatment guidelines, and on the important role of patient preferences in treatment decisions.

These two issues, a lack of epidemiological data for Switzerland and the promotion of the patient-perspective in MS research, triggered the Swiss MS Society to establish a novel MS registry. The Swiss MS Registry (SMSR) not only contributes basic data on the epidemiology of MS in Switzerland, through its patient-centered design it can also fill an important niche in national and international research together with other studies [5, 6]. This manuscript describes the main philosophy behind the SMSR and elaborates on the study design, as well as the methods and contents of the data collection. Moreover, a companion paper illustrates how the SMSR merges traditional with novel, internet-driven research approaches to leverage the advantages of both while mitigating legal, ethical, and data security risks [7].

## Methods/Design

### Study objectives

The SMSR was established with three primary goals. First, the SMSR shall provide the basis for more accurate prevalence estimates and long-term monitoring of epidemiological trends of MS in Switzerland.

Second, the SMSR will establish a study base for patient-centered MS research in Switzerland, thereby focusing on assessments of the disease burden for persons with MS (PwMS) and their relatives and proxies. Along the same lines, the SMSR aims to contribute research to previously understudied topics concerning patients’ life circumstances and experiences (e.g. MS and work), as well as on clinical and health care related aspects (e.g. access to specialized MS care).

Third, by creating a versatile database and a flexible study infrastructure, the SMSR offers a platform for nested investigations. From the outset, the SMSR aimed to be an interdisciplinary, open, collaborative project, designed to leverage other existing research efforts. In particular, by systematic analysis of the Swiss and the international MS research landscape [5], knowledge gaps were identified and subsequently taken into account during the study planning phase. Examples of such topics include health-related quality of life, alternative medicines, physiotherapy, or work and insurance, which have now become part of the SMSR core data collection. Furthermore, the SMSR data structures were designed for compatibility with ongoing national and international collaborations (EUReMS) [8]. Specifically, the SMSR has established a strategic partnership with the clinically-oriented Swiss MS Cohort (SMSC) study [9] and strives to complement the SMSC data collection by adding further data from the patients’ perspectives on diagnosis, treatment, and general well-being. In both studies, informed consent documents include opt-in agreements to allow future data exchanges between the SMSR and the SMSC for data validation and research projects.

### Study design

The SMSR is an open-ended, longitudinal and prospective, nationwide, patient-centered study (http://www.ClinicalTrials.gov identifier: NCT02980640). It follows a citizen-science approach with direct involvement of PwMS, who contribute to the SMSR development and research via representation in the Scientific Assembly.

Unlike other MS registries, the data are primarily collected directly from participants via structured questionnaires, that is, without intermediary health professionals. Consequently, surveys must be understandable by lay-persons and be provided in the three main Swiss official languages (German, French, Italian). Participation is offered via an online web system and as paper & pencil versions.

Design and the research strategy of the SMSR are based on three major tenets, which were flexibility regarding study contribution by PwMS (from one-time to longitudinal participation), provision of data feedbacks to participants and regular information on how data are being used, as well as the involvement of PwMS in study design and execution.

The SMSR includes four participation options for PwMS: (1) they can simply fill in a one-time survey, (2) additionally they can complete regular, semi-annual surveys on varying topics, (3) when signing the informed consent, they can decide to grant access to their medical records for abstraction by the SMSR, and (4) participants can allow the SMSR to exchange their data with the SMSC. Combinations of these options are possible. The decision for participation in either study module needs to be taken at time to informed consent signature (layer 1) on an opt-in basis but can be revised later, for example if a person wishes to withdraw her/his consent for medical record release.

**Fig. 1:**
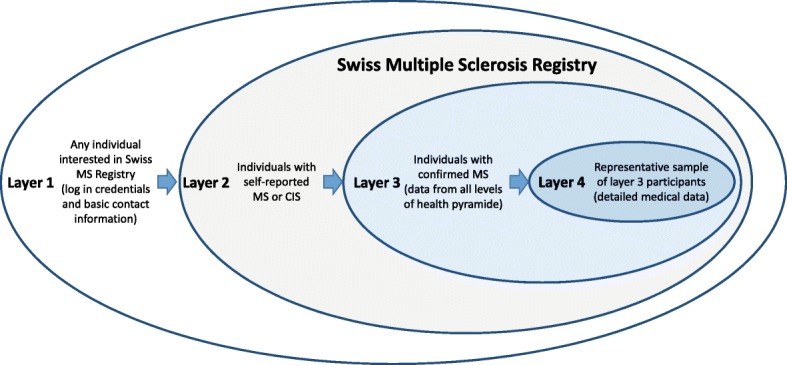
Study design of the Swiss Multiple Sclerosis Registry (SMSR)

These different participation forms are also reflected (and accommodated) in the SMSR study design, which entails different layers (Fig. 1) through which participants can gradually navigate. This layer concept was inspired by the amyotrophic lateral sclerosis registry in the USA [10]. The first, outermost layer 1 includes the deposition of contact information and informed consent signature. During these steps, prospective participants can also decide on which study modules they would like to contribute to.

The SMSR registration process is considered complete upon submission of a first, one-off questionnaire, which also demarks the second registry layer. The purpose of layer 2 is to collect basic demographic and medical data from as many PwMS as possible for epidemiological investigations (e.g. on MS prevalence in Switzerland).

In layer 3, the SMSR further offers regular participation by means of semi-annual surveys. The main objective of this third layer is a longitudinal data collection on disease burden, MS progression, physical and mental well-being, and changing life circumstances (Table 1). Layer 3 is open to all participants who have completed layer 2 and are willing to submit a confirmation of their diagnosis, signed by their treating physician.

**Table 1:** Overview of assessments in the Swiss Multiple Sclerosis Registry

Topics	Layer 2 (one-off Survey)	Layer 3 Baseline (one-off Survey)	Layer 3 Follow-up 1 (every 6 months)	Layer 3 Follow-up 2 (every 12 months)	Layer 4 (Medical chart review every 12 months)
Sociodemographic variables					
Personal information	X	X		X	
Family and living situation	X	X		X	
Education, profession		X		X	
Working situation		X		X	
Occupational changes due to MS		X		X	
Societal context (e.g. disability insurance)		X		X	
Disease course					
First symptoms	X				X
Symptoms ever, current	X	X	X	X	X
Symptoms changes (prior and after diagnosis)					X
Disease stage, type of MS	X	X	X	X	X
EDSS-score		X	X	X	X
Mobility		X		X	
Relapses		X	X	X	X
Age of disease progression		X			
Diagnostic process					
Age at first symptoms	X				
Time of first medical doctor visit			X		
Personal experience of diagnostic process			X		
Treatment					
Disease modifying treatment ever/current	X	X	X	X	X
Non-drug therapies	X	X	X	X	X
Side effects / adverse events	X	X	X	X	X
Therapy stop / interruption	X	X	X	X	
Interventions against side effects		X	X	X	X
Alternative medicine	X	X	X	X	
Additional medicine / supplements	X	X	X	X	
Cannabis treatment		X	X	X	
Comorbidities					
Comorbidities		X	X	X	X
Medication for comorbidities		X	X	X	X
Risk factors and family history					
Weight		X		X	
Smoking behavior (and exposure)		X		X	
Alcohol consumption		X		X	
Nutrition	X			X	
Previous medical history		X			
Childhood illnesses		X			
Vaccination		X		X	
MS family history	X				X
Sun exposure		X			
Hormonal factors (only women)		X			
Nutrition and Lifestyle					
Nutrition change since diagnosis	X			X	
Lifestyle change since diagnosis	X			X	
Physical Activity	X	X		X	
Care and medical aids					
Institutions visits	X	X		X	
Care types		X		X	
Contact with healthcare professionals	X	X		X	
Specialists consultation	X	X		X	
Confidence in specialists		X		X	
Medical aids		X		X	
Domestic assistance		X		X	
Housework		X		X	
Disclosure of MS		X		X	
Quality of Life					
Health related quality of life (EQ-5D-5 L;WHO 5-item well-being index)	X	X	X	X	
Mental health					
Psychological well-being	X	X	X	X	
Depression		X	X	X	
Burden of disease					
Individual burden (e.g. symptoms,...)	X	X	X	X	
Societal burden		X	X	X	
Economic burden				X	

The self-reported data from layers 1 to 3 are complemented by clinical data in layer 4, which stems from two sources. Participants may either elect to release their patient charts for medical record abstraction or they can allow data exchanges between the SMSR and the SMSC in case of dual participation in both studies. These clinical layer 4 data are of critical importance for validation of self-reported medical events such as symptoms, relapses, comorbidities, and treatments. The goal is to document up to 1000 participants, and the patient selection for layer 4 medical record abstraction is made by the SMSR study center based on age, gender, disease stage, and treatment setting in order to ensure representativeness.

Layers 3 and 4 also allow data collections for specific projects, which can either be included into regular follow-up surveys and data abstractions or be conducted as separate nested measurements outside the regular follow-up schedule (but possibly requiring its own ethical approval / informed consent). Combined, the layer design offers a very flexible structure, which not only accommodates specific research needs, but also offers participants a choice of different commitment levels.

### Study population

The SMSR is open to all adults aged 18 years and older who were diagnosed with MS or a clinically isolated syndrome (CIS) and who live or are regularly receiving medical care in Switzerland. For contribution to layers 3 and 4, the MS/CIS diagnosis needs to be confirmed by a physician.

### Study recruitment

The SMSR was initiated by and developed in close collaboration with the MS community in Switzerland, represented by the Swiss MS Society. The SMSR is executed by the University of Zurich and promoted as a project of the Swiss MS Society. The Society’s extensive network and various media outlets (website, member magazine, social media activities, newsletters) allowed a quick and nationwide dissemination of information about the SMSR to potentially interested participants, as well as physicians.

Registry enrolment occurs by means of self-recruitment and peer referral. Clinics and private practices are involved in recruitment insofar as they provide postcards and leaflets about the SMSR and raise awareness of the Registry’s existence among their patients. For SMSR enrolment, interested persons can either send in a postcard to the SMSR data center, upon which they will receive additional instructions for joining the SMSR. Alternatively, they can login directly to a website (www.ms-register.ch), create an account, and - after signing the informed consent- access the surveys.

### Ethical aspects

The study has been approved for nationwide conduct as a multi-centric study by the Ethics Committee Zurich (Study number PB-2016-00894). Informed consent is obtained from all participants, either electronically upon first access to the SMSR platform or as part of the registration process for participants who prefer paper & pencil versions. In line with the SMSR’s strategy to offer various commitment levels, the informed consent offers three opt-in choices regarding study module participation.

The first module involves the diagnosis confirmation, which is prerequisite for participation in regular follow-up surveys (layer 3) and medical record abstraction (layer 4). That is, refusal of providing permission of the diagnosis confirmation by the treating physician implies that only participation in layer 2 is possible.

The second module is the medical record abstraction. Giving approval to this study option means providing the SMSR data center personnel access to medical records. Prior to first record access, participants are notified and given 3 weeks to notify the SMSR data center in case they wish to reconsider their decision.

Third, SMSR participants can agree to share their data with the SMSC study and vice versa. This study module only applies to persons enrolled in both studies. Overall, agreement to these three study modules is in excess of 90%. Withdrawal from any of these study modules or from SMSR participation is possible at any time and without provision of explanations, but very few persons have chosen to do so (< 20 participants).

### Data acquisition

Data are collected directly from participants via structured questionnaires. The entry questionnaire, which constitutes layer 2, takes approximately 20 min to complete and collects data on a person’s MS history, symptoms, treatments, diagnosis, risk factors, as well as changes in lifestyle behavior due to MS (Table 1). Approx. 75% of all layer 2 participants also contribute to the regular surveys in layer 3. These semi-annual questionnaires require 45 min and collect data on recent medical events, drug- and non-drug treatments, living and familial situation, work and evolving special topics (e.g. on patients’ experiences of the diagnostic process). Participants are either informed via email when a new questionnaire is ready or, in case of participation on paper, the survey is mailed directly, along with a pre-stamped return envelope. The online system implements completeness and plausibility checks, as well as bifurcations if questions pertain only to a subgroup of respondents. It further allows users to pause the entry process and to store intermediary results. Upon completion and submission of the questionnaire, the answers are stored in the study database and can no longer be changed or updated by the participant. A help desk, located at the SMSR data center, is available via phone and email in case of questions.

For all layer 4 data collections, participants must have either agreed to release their medical records or to exchange data with the SMSC in case of dual enrolment. The SMSR data center manages all data collections and exchanges. Medical record abstraction is performed on site at clinics and private practices on an annual basis.

### Technical aspects

The SMSR has established an online platform for survey delivery and data collection, which also includes a patient diary, as well as features for participants to analyze their own data. The data collection platform was developed in close collaboration with the S3IT (Science and IT) Division of the University of Zurich. The backend and the frontend applications are hosted on secure servers at the University. The backend consists of a Mongo Database and a set of Python scripts providing API functions for the frontend. The frontend is programmed in Java Script Angular by a dedicated Web Developer and also includes a Content Management System for simple creation and update of online study forms.

Security and data safety are of great concern. Safety measures include e-mail confirmation during account creation (double opt-in), password strength enforcement, 256-TLS secured SSL communication between clients and servers, as well as strict separation of identifiable and research data. For user administration and access authentication, a single sign-on system was developed (Mysql, PHP), which is hosted outside the University of Zurich by a certified Swiss Internet provider. All identifiable data are encrypted and IT security was assessed by an external, specialized company.

### Measurements

**Fig. 2:**
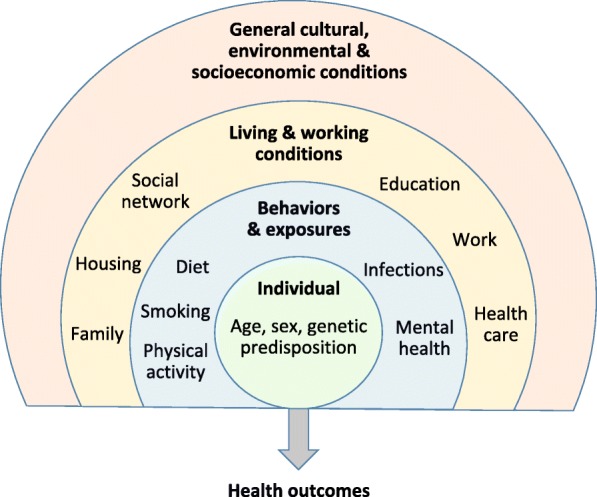
Holistic ascertainment of determinants of health outcomes in individuals affected by MS

One particular challenge for studying MS is its multifaceted manifestation. Therefore, in line with its patient-centered design, the SMSR data collection goes well beyond medical information also including data on sociodemographic factors, family history, (lifestyle) behaviors and exposures, risk factors, patient-reported outcomes and living and working conditions (Fig. 2). In addition, data on the general cultural, environmental and socioeconomic conditions can be added based on where participants live. The breadth of data collection enables comprehensive study questions on disease burden, causes of disease, prognostic questions, therapeutic questions as well as research on health services for PwMS. The following paragraphs and Table 1 provide further details on the specific topics, as well as their assessment frequency.

#### Symptoms, relapses and progression

Data on symptoms and MS progression are collected by self-reporting in regular surveys. At baseline, participants are instructed to indicate all symptoms they have ever experienced, the first MS symptoms they ever noticed, as well as symptoms that have occurred during the past 6 months. The questionnaire further includes items on the number of relapses and corticosteroid treatments (including treatment settings or, where applicable, reasons for absence of treatment initiation). This information is updated on an annual basis in layer 3 and validated with clinical layer 4 data where available. Aspects of mobility are queried in layer 3 by use of the validated MS Questionnaire for Physiotherapists (MSQPT) [11].

#### Treatments, medication and adherence to disease-modifying treatment (DMT)

In the layer 2 survey, participants are asked to list all DMTs they have taken in the past 6 months, as well as all DMTs that were ever taken. The survey also collects basic data on alternative medications and non-pharmacological drug treatments. These data are updated semi-annually in regular layer 3 questionnaires. Adherence to DMT is assessed at least annually in layer 3 on the basis of a short questionnaire that queries the number of missed doses over the past 4 weeks.

#### Health-related quality of life

The EuroQol- 5 Dimension (EQ5D) instrument is assessed on an annual basis [12, 13], for the first time in layer 2. The EQ5D is also integrated in the patient diary, which is accessible through the online data collection platform. The regular follow-up questionnaires also contain the Warwick-Edinburgh Scale [14] and the World Health Organization (WHO) 5-item well-being scale, which are also implemented annually [15]. In addition, a validated single-item questionnaire on (positive) health behavior is included [16].

#### Mental health, depression, and fatigue

In order to assess fatigue, which represents one of the most prominent symptoms of MS, the Modified Fatigue Impact Scale (MFIS-21) is applied [17, 18]. In addition, the well-established Multiple Sclerosis Impact Scale (MSIS-29) is assessed to address physical and psychological impact from the patient perspective in the last 2 weeks. [19]

Additional questions covering aspects of mental health and depression are assessed in specialized follow-up surveys by applying the standardized Beck Depression Inventory (BDI)-Fast Screen [20], and Mini SPIKE [21, 22].

#### Circumstances of life

The layer 3 questionnaire further includes a series of questions on education, living situation, work, disability insurance, social support (“Oslo 3 Social Support Scale” [23]) and benefit finding (“Benefit Finding in MS Scale” (BFiMSS)) [24]. These questions were developed in close collaboration with the Swiss MS Society, neuropsychologists, and PwMS. The work history and insurance status are updated annually.

### Statistical analyses

The SMSR Data Center, located at the Epidemiology, Biostatistics and Prevention Institute of the University of Zurich, is responsible for data collection and quality assurance. Statistical analyses are performed continuously for monitoring purposes, for data feedbacks to study participants, and for specific scientific projects. One of the SMSR core aims is to establish a long-term, epidemiological monitoring of MS in Switzerland. As no single study is able to establish precise prevalence estimates for MS in Switzerland, the SMSR approaches this goal by combining information from different sources (e.g. the SMSC) to estimate the total size of the Swiss MS population. Thereby, the SMSR employs methods of sensitivity analysis [25], data triangulation [26], and individual-based mathematical modelling (e.g. [27]) in order to refine these estimates and assess the validity of results.

### Study status

Since its launch on June 25, 2016, the SMSR has enrolled 1605 persons with MS who have signed the informed consent (status per September 27, 2017). Recruitment is ongoing, with 1–2 new enrolments daily. In total, 1343 persons have successfully completed the first layer 2 questionnaire. Moreover, 1005 persons also opted to participate in regular surveys and have successfully completed the first layer 3 questionnaire, which is the starting point for semi-annual follow-up surveys. Of those 1005 persons, 833 have already submitted a diagnosis confirmation. Overall, 80% of participants complete the questionnaires online, the remainder on paper.

### Organization and funding

The SMSR was established at the initiative of the Swiss MS Society, which is also the financial sponsor of the study. The SMSR has a clearly defined governance structure and guidelines in place to ensure a cooperative, transparent operation. The main steering body of the SMSR is the Scientific Assembly, which oversees the research strategy, elects the SMSR president, and grants access to research data. It consists of elected representatives of major MS centers, the Swiss MS Society, the MS research community, neurorehabilitation clinics, and, last but not least, PwMS. Scientific Assembly members bring in diverse professional expertise such as neurology, nursing, physiotherapy, neuropsychology, IT, or neuroimmunology. The Scientific Assembly is structured into three thematic research committees, which oversee the operational aspects of the MS registry development and initiate research projects in their respective area of expertise. The first Research Committee is concerned with all patient and population-based research activities (including review of questionnaires and research instruments). A second Research Committee deals with all things related to IT, databases, and analysis of unstructured data. This IT Research Committee also initiates and develops technology-driven registry extensions. The third Research Committee oversees all clinical and laboratory research and has the lead in the development of the layer 4 data collection.

Anonymized SMSR data are - in principle – available to all qualified researchers for specific projects. Guidelines regulate procedures for data access, which consist of the submission of a research proposal to the Scientific Assembly. The proposal is then scored according to feasibility, scientific soundness, and alignment with overarching SMSR goals and examined by independent reviewers before being approved by the members of the Scientific Assembly. PwMS and the Swiss MS Society are also involved in this approval process.

## Discussion

The SMSR is a longitudinal, patient-centered study, designed to fill important knowledge gaps on MS epidemiology and disease burden for patients in an environment where systematic compilation of routine data from providers and/or insurers is not feasible. The close collaboration with a patient-organization, PwMS, and MS researchers has created a unique approach and philosophy for data collection and research. In particular, the SMSR stands out for its participatory approach to registry development and research, a singular blend of patient-reported and clinical data, as well as a transparent governance.

Other web-based, patient-centered initiatives include, for example, the UK MS Register, which is a nationwide, web-based study for the UK [6], which also has participatory elements [28]. The UK Register gathers basic information on MS status and progression, socio-demographic factors, and a number of several patient-reported instruments and has recruited over 11,000 participants in 5 years [6, 29]. A second notable example is the Dutch MS Study, which also collects longitudinal data on quality of life and health status by means of validated, patient-reported instruments [30]. Although comparable in scope and methodology, we believe that the SMSR offers additional noteworthy features.

First, the SMSR pursues a participatory approach to study planning and research. PwMS and the Swiss MS Society are active contributors to the SMSR, which enhances motivation for participation and greatly improves the clarity and structure of the surveys, as well as communication of the findings.

Second, the layer model offers several participation options with different time commitments. The SMSR strives to obtain basic epidemiological data from as many PwMS as possible by means of a short one-off survey. Participants may also opt to contribute to semi-annual surveys, which are somewhat more time-demanding. Other additional study modules such as medical record abstraction come without any additional workload for patients and care providers, however.

Third, the SMSR offers regular data feedbacks for private use and self-monitoring. Feedbacks are contextualized in the whole population of study participants. That is, participants receive additional information on whether their specific survey response is rare or common among the other participants. Data feedbacks are also a very efficient means to solicit data corrections of key variables from participants.

Fourth, the SMSR has established a governance structure that involves PwMS via representation. The Scientific Assembly determines the scientific strategy and approves data requests for research. The inclusion of patient representatives and the Swiss MS Society in governing bodies guarantees influence of the MS patient community over project guidelines, research agenda and data usage.

Finally, by combining traditional and novel, web-based data collection methods the SMSR exploits the potential of societal and technological trends while mitigating legal and ethical risks. By offering different participation modes (web, paper & pencil) and in the three main national languages, the SMSR leverages benefits of modern technology for those who wish to participate online, without leaving less internet-savvy persons behind. Furthermore, unlike commercial initiatives, the SMSR operates under clear regulations and guidelines (Swiss Human Research Law), which guarantee the rights of participants.

Therefore, while the SMSR may not be the first participatory study in the field of MS research to take advantage of technical innovations, in particular by providing a web portal for data collection and visualization, it clearly takes patient participation to a new level, thereby distinguishing itself from other international MS registries.

## Conclusion

The SMSR is an innovative, prospective study that combines advantages of traditional and novel research methods in medical research. Moreover, the SMSR is unique by taking the participatory approach further than any other study in the MS field and by covering a large variety of domains that are relevant to PwMS’ experiences and life circumstances. What is more, the flexible IT infrastructure and clear governance rules support extensions to the core protocol and data collections, thus creating a future-oriented platform that is well suited to respond to future technological innovations and research trends, but also to challenges in health care provision in MS.

## Abbreviations

BDI: Beck Depression Inventory
CIS: Clinically isolated syndrome
DMT: Disease-modifying treatment
EQ5D: EuroQol- 5 Dimension
IT: Information technology
MS: Multiple sclerosis
MSIS-29: Multiple Sclerosis Impact Scale
MSQPT: Multiple sclerosis questionnaire for physiotherapists
PwMS: Person with MS
SMSC: Swiss Multiple Sclerosis Cohort Study
SMSR: Swiss Multiple Sclerosis Registry
WHO: World Health Organization
